# Comparison of Five Methods for the Determination of Rubella Immunity

**DOI:** 10.1155/S1064744994000062

**Published:** 1994

**Authors:** Robert L. Sautter, Arthur E. Crist, Lynn M. Johnson, William D. LeBar

**Affiliations:** 1 Department of Pathology Harrisburg Hospital Polyclinic Medical Center Harrisburg PA USA; 2 Department of Pathology Polyclinic Medical Center Harrisburg PA USA; 3 Department of Laboratories Polyclinic Medical Center Harrisburg PA USA; 4 Citation Clinical Laboratories Providence Hospital Microbiology Laboratory Southfield MI USA; 5 Clinical Microbiology Department of Pathology Harrisburg Hospital South Front Street Harrisburg PA 17101-2099 USA

## Abstract

*Objective:* The purpose of this study was to compare the accuracy of commonly used methods for the detection of rubella immunity, especially the fully automated IMx assay.

*Methods:* A total of 190 sera (101 immune and 89 non-immune) submitted to Harrisburg Hospital or Polyclinic Medical Center for the determination of rubella immunity were tested by enzyme immunoassay (IMx and Rubazyme, Abbott Diagnostic Laboratories, North Chicago, IL), indirect immunofluorescence (FIAX, Whittaker Bioproducts, Walkersville, MD), and latex agglutination (Rubascan, Becton Dickinson Microbiology Systems, Cockeysville, MD, and Rubalex, Wellcome Diagnostics, Research Triangle Park, NC). Specimens were frozen at –30℃ until the study was initiated. Each of the assays was performed according to the manufacturers' specifications. Sensitivity, specificity, accuracy, and positive and negative predictive values for each assay were calculated using a consensus result of the 5 methods tested.

*Results:* The sensitivity, specificity, and accuracy, respectively, of the test systems were as follows: IMx, 96%, 97%, and 96%; Rubazyme, 100%, 99%, and 99%; Rubascan, 100%, 98%, and 99%; Rubalex, 99%, 97%, and 98%; and FIAX 90%, 100%, and 95%. False negative reactions were seen with the FIAX system.

*Conclusions:* The IMx system, a new “walk away” system from Abbott Diagnostic Laboratories and the Rubazyme systems performed well; however the latex agglutination tests proved to be the most rapid and convenient methods for screening sera for the presence of rubella immunity.


Infectious Diseases in Obstetrics and Gynecology 1:188–192 (1994)

© 1994 Wiley-Liss, Inc.

### Comparison of Five Methods for the Determination of Rubella Immunity

**Robert L. Sautter, Arthur E. Crist, Jr., Lynn M. Johnson, and William D. LeBar**

*Department of Pathology, Harrisburg Hospital (R.L.S.), Departments of Pathology (A.E.C.) and Laboratories (L.M.J.), Polyclinic Medical Center, Harrisburg, PA, and Citation Clinical Laboratories, Providence Hospital (W.D.L.), Microbiology Laboratory, Southfield, MI*

### Abstract

***Objective:*** **The purpose of this study was to compare the accuracy of commonly used methods for the detection of rubella immunity, especially the fully automated IMx assay.**

***Methods:*** **A total of 190 sera (101 immune and 89 non-immune) submitted to Harrisburg Hospital or Polyclinic Medical Center for the determination of rubella immunity were tested by enzyme immunoassay (IMx and Rubazyme, Abbott Diagnostic Laboratories, North Chicago, IL), indirect immunofluorescence (FIAX, Whittaker Bioproducts, Walkersville, MD), and latex agglutination (Rubascan, Becton Dickinson Microbiology Systems, Cockeysville, MD, and Rubalex, Wellcome Diagnostics, Research Triangle Park, NC). Specimens were frozen at −30°C until the study was initiated. Each of the assays was performed according to the manufacturers' specifications. Sensitivity, specificity, accuracy, and positive and negative predictive values for each assay were calculated using a consensus result of the 5 methods tested.**

***Results:*** **The sensitivity, specificity, and accuracy, respectively, of the test systems were as follows: IMx, 96%, 97%, and 96%; Rubazyme, 100%, 99%, and 99%; Rubascan, 100%, 98%, and 99%; Rubalex, 99%, 97%, and 98%; and FIAX 90%, 100%, and 95%. False negative reactions were seen with the FIAX system.**

***Conclusions:*** **The IMx system, a new "walk away" system from Abbott Diagnostic Laboratories and the Rubazyme systems performed well; however the latex agglutination tests proved to be the most rapid and convenient methods for screening sera for the presence of rubella immunity.**
**© 1994 Wiley-Liss, Inc.**

Key words

IMx, enzyme immunoassay, latex agglutination, fluorescent immunoassay, comparison of viral immunological methods

Public health measures to reduce the transmission of rubella are dependent upon the vaccination of children ≥12 months of age, school-age children not previously immunized, and susceptible adults. The occurrence of rubella during pregnancy can result in severe congenital abnormalities of the newborn. Current guidelines suggest that women of childbearing age should be evaluated for immune status to rubella. If they are negative, they should be monitored throughout the pregnancy for seroconversion. If these women remain negative following delivery, then they should be vaccinated postpartum. In addition, health care personnel are monitored at most pre-employment physicals; if they are found to be non-immune, vaccination is recommended. The rubella antibody test is one of the most frequently ordered tests in the serology laboratory.

**Address correspondence/reprint requests to Dr. Robert L. Sautter, Director, Clinical Microbiology, Department of Pathology, Harrisburg Hospital, South Front Street, Harrisburg, PA 17101-2099.**

Received November 29, 1993
Accepted January 28, 1994

Basic Science Article

In the past, the recommended method for determining immune status to rubella was the hemagglutination inhibition (HI) test. For the most part, HI has been replaced with less cumbersome methods, including passive hemagglutination,[1–5] latex agglutination,[1–13] fluorescenceimmunoassay,[1–4,6,14,15] enzyme immunoassay,[1,2,4,5,10,11,15–18] and radial hemolysis in gel.[4,13] These methods have been shown to be as accurate as HI and in some cases more sensitive, specific, and reproducible.[5,8,9,11]

Recently, a fully automated IMx immunoassay analyzer was developed for the detection of IgG and IgM antibodies to rubella virus.[16,19] A fully automated system provides for a more objective result, decreases the technical time and cost required to perform the assay, offers standardization of the test procedure, and lends itself to physician office testing. The purpose of this study was to compare several commercially available methods for the determination of rubella immunity, including the fully automated IMx immunoassay analyzer.

### MATERIALS AND METHODS

### Specimens

A total of 190 serum specimens submitted to the clinical microbiology laboratory at Harrisburg Hospital and Polyclinic Medical Center were tested by 5 methods. The serum samples were obtained from specimens submitted for rubella serology. Sera were tested on receipt in the laboratory by fluorescence immunoassay (FIAX, Whittaker Bioproducts, Waldersville, MD) and latex agglutination (Rubascan, Becton Dickinson Microbiology Systems, Cockeysville, MD) and then were stored at −30°C until subsequent testing. The stored sera remained frozen for 6 months and then were tested using the remainder of the assays.

### IMx

The IMx (Abbott Diagnostic Laboratories, North Chicago, IL) is an automated procedure based on microparticle enzyme immunoassay technology. Once the serum sample is placed into the reaction cell of the carousel, all additional steps are performed by the instrument. The principle and operation of the IMx have been described previously[16,18] and are reviewed briefly here. Once controls and samples have been loaded onto the carousel and the instrument started, the probe/electrode assembly delivers the sample and diluent buffer to the predilution well of the reaction cell. Next, the microparticles coated with rubella virus and an aliquot of the diluted sample are added to the incubation well. If antibodies to rubella are present in the patient's serum, they will bind to the antigen-coated microparticle forming an antigen-antibody complex. Diluent buffer is added to the reaction mixture, and an aliquot of the antigen-antibody complex is added to the glass fiber matrix. The microparticles bind irreversibly to the glass fiber matrix. The matrix is washed to remove unbound material, and an alkaline phosphatase-conjugated anti-human IgG is dispensed onto the matrix and binds to the antigen-antibody complex. The matrix is then washed again to remove unbound material. The substrate 4-methylumbelliferyl phosphate is then added to the matrix, and the fluorescent product formed is measured by the optical assembly of the instrument. Those specimens that exhibited values ≥10 IU of IgG antibody to rubella virus were considered immune. At the beginning of the study, 6 calibrators were run to establish a calibration curve. Once established, this calibration curve has been shown to be stable for at least 2 weeks. Positive and negative controls were included in each run.

### FIAX

Fluorescence immunoassay was performed using Rubella G kits supplied by the manufacturer. The method was performed according to the manufacturer's instructions and has been described previously.[15] A result lower than 8 indicated susceptibility to rubella and a result greater than 12 indicated immunity. An equivocal zone of 8–12 has been established by the manufacturer to avoid false positive readings, and serum specimens in this range were retested.

### Latex Agglutination

Latex agglutination was performed using the Rubascan and Rubalex (Wellcome Diagnostics, Research Triangle Park, NC). Both procedures were tested according to the manufacturers' instructions and have been described previously.[2,6] Serum specimens tested with the Rubalex method were diluted 1:10 prior to testing. With the Rubascan method, serum samples were tested undiluted and diluted 1:10 as recommended by the manufacturer.

TABLE 1. Performance characteristics (%) of the 5 methods for determining rubella immune status

| Method | Sensitivity | Specificity | PPV | NPV | Accuracy |
|---|---|---|---|---|---|
| IMx | 96 (97/101) | 97 (86/89) | 96 (97/101) | 96 (86/90) | 96 (183/190) |
| FIAX[a] | 90 (89/99)* | 100 (89/89) | 100 (89/89) | 90 (89/99) | 95 (178/188) |
| Rubascan | 100 (101/101) | 98 (87/89) | 98 (101/103) | 100 (87/87) | 99 (188/190) |
| Rubalex | 99 (100/101) | 97 (86/89) | 97 (100/103) | 99 (86/87) | 98 (186/190) |
| Rubazyme | 100 (101/101) | 99 (88/89) | 99 (101/102) | 100 (88/88) | 99 (189/190) |

[a]Two specimens gave equivocal results using the FIAX method and were elminated from the comparison.
*$P = 0.0008$.

TABLE 2. Samples with discrepant results

| IMx | FIAX | Rubascan | Rubalex | Rubazyme | Final interpretation | No. of samples |
|---|---|---|---|---|---|---|
| Negative | Negative | Positive | Positive | Negative | Negative | 2 |
| Positive | Negative | Positive | Positive | Positive | Positive | 4 |
| Positive | Equivocal | Positive | Positive | Positive | Positive | 2 |
| Negative | Negative | Positive | Positive | Positive | Positive | 4 |
| Positive | Negative | Negative | Negative | Negative | Negative | 2 |
| Positive | Positive | Positive | Negative | Positive | Positive | 1 |
| Negative | Negative | Negative | Positive | Negative | Negative | 1 |
| Positive | Negative | Negative | Negative | Positive | Negative | 1 |

### Enzyme Immunoassay (EIA)

EIA was performed using Rubazyme (Abbott Diagnostic Laboratories) kits supplied by the manufacturer. The method was performed and the results were interpreted according to the manufacturer's instructions and have been described previously.[5] Each serum sample was tested in duplicate.

### Comparison of Results and Statistical Analysis

Specimens were considered positive or negative for rubella antibody when 3 or more methods were in agreement. Each assay method was compared to the "consensus" result in order to determine sensitivity, specificity, accuracy, and predictive values of the method. Statistical analysis and evaluation of the 5 methods were made using the chi-square test (StatView + Graphics, Abacus Concepts, Inc., Berkeley, CA).

### RESULTS

Using a consensus of 3 or more methods, we found a total of 101 specimens to be positive for rubella antibody and 89 specimens to be negative. Performance characteristics of the methods for the detection of rubella antibody can be seen in Table 1.

The FIAX, with a sensitivity of 90%, was the least sensitive of the methods evaluated ($P = 0.0008$). The other 4 methods were comparable in sensitivity, which ranged from 96% to 100%. The specificity, positive predictive value (PPV), negative predictive value (NPV), and accuracy of the 5 methods were as follows: 97%, 96%, 96%, 96% for IMx; 100%, 100%, 90%, 95% for FIAX; 98%, 98%, 100%, 99% for Rubascan; 97%, 97%, 99%, 98% for Rubalex; and 99%, 99%, 100%, 99% for Rubazyme. There was no statistically significant difference in specificity in the 5 methods ($P = 0.3158$).

Data on samples giving discrepant results are found in Table 2. There were 17 samples that gave discrepant results in assays. Four samples were negative, and 2 were equivocal (even on repeat testing) by the FIAX method but positive by the other 4 methods. Four additional samples were negative by FIAX and IMx but positive by all other methods. The other 7 discrepancies were distributed among the other assays.

### DISCUSSION

The Advisory Committee for Immunization Practices of the Center for Disease Control, Atlanta,

GA, has recommended that a positive result by any test for rubella antibody be accepted as evidence of rubella immunity.[20] In support of this recommendation are several studies that have shown that low antibody titers, particularly those detected by latex agglutination, are sufficient to protect against infection with attenuated virus.[8,12] The latex agglutination methods (Rubascan and Rubalex), as demonstrated in this study, are as sensitive, specific, and accurate as other methodologies. They also have the advantages of requiring no pretreatment of serum samples, small sample volume, rapid turnaround time (less than 10 min), and no purchase of capital equipment.

The 3 remaining assays are either fully automated (IMx) or semiautomated (FIAX and Rubazyme). Previous studies on the fully automated IMx rubella IgG assay have shown that the system is more sensitive than and as specific as conventional EIA technology.[16,18] Schaefer et al.[18] showed that the IMx had a sensitivity of 99.9% compared to a sensitivity of 96.5% for Rubazyme. Specificity was identical for both assays at 98.9%. Similar results were obtained by Abbott et al.[16] and Skurrie et al.[19] In these previous studies, the IMx was only compared to conventional EIA with discordant results referred by passive hemagglutination. Other commercially available systems such as latex agglutination and fluorescent immunoassay were not evaluated. In the present study, the IMx was found to be more sensitive and accurate than fluorescent immunoassay but equivalent to the latex agglutination assays and conventional EIA.

The primary purpose of rubella screening is to identify non-immune women of childbearing age. Therefore, a false positive result would be an error with the most serious consequences since women who are truly non-immune would not be vaccinated and would be fully susceptible to rubella infection. Subsequent infection during pregnancy would pose a risk to the unborn fetus. On the other hand, a false negative result, although not as serious, could result in potential morbidity to the patient due to unnecessary vaccination. The FIAX method showed the lowest diagnostic accuracy (95%), primarily due to a 10% false negative rate. The other methods evaluated had diagnostic accuracies ranging from 96% to 99%, with few false positive or false negative results. On the basis of the results presented here, all of the methods evaluated, with the possible exception of the FIAX assay, can be used reliably to determine rubella immune status.

### ACKNOWLEDGMENTS

This work was funded in part by Carter Wallace, Inc., Cranbury, NJ.

### REFERENCES

1. Castellano GA, Madden DL, Hazzard GT, et al.: Evaluation of commercially available diagnostic test kits for rubella. J Infect Dis 143:578–584, 1981.
2. Chernesky MA, DeLong DJ, Mahony JB, Castriciano S: Differences in antibody responses with rapid agglutination tests for the detection of rubella antibodies. J Clin Microbiol 23:772–776, 1986.
3. Fayram SL, Akin S, Aarnaes SL, Peterson EM, de la Maza LM: Determination of immune status in patients with low antibody titers for rubella virus. J Clin Microbiol 25:178–180, 1987.
4. Herrmann KL: Available rubella serologic tests. Rev Infect Dis 7(Suppl 1):108–112, 1985.
5. Kleeman KT, Kiefer DJ, Halbert SP: Rubella antibodies detected by several commercial immunoassays in hemagglutination inhibition-negative sera. J Clin Microbiol 18: 1131–1137, 1983.
6. Fayram SL, Nakasone A, Aarnaes S, Zartarian M, Peterson EM, de la Maza LM: Fluorescence immunoassay and passive latex agglutination as alternatives to hemagglutination inhibition for determining rubella immune status. J Clin Microbiol 17:685–688, 1983.
7. Freeman S, Clark L, Dumas N: Evaluation of a latex agglutination test for detection of antibodies to rubella virus in selected sera. J Clin Microbiol 18:197–198, 1983.
8. Grangeot-Keros L, Badur S, Pons JC, Duthu S, Pillot J: Use of in vivo challenge to assess rubella immunity determined by hemagglutination inhibition and latex agglutination. Res Virol 140:437–442, 1989.
9. Leon P, deOry F, Domingo C, Lopez JA, Echevarria JM: Evaluation of a latex agglutination test for screening antibodies to rubella virus. Eur J Clin Microbiol Infect Dis 7:196–199, 1988.
10. Simor AE, Chua R, Low DE: Evaluation of a new latex test and a new enzyme immunoassay for determination of rubella immunity. J Clin Microbiol 26:1582–1583, 1988.
11. Steece RS, Talley MS, Skeels MR, Lanier GA: Comparison of enzyme-linked immunosorbent assay, hemagglutination inhibition, and passive latex agglutination for determination of rubella immune status. J Clin Microbiol 21:140–142, 1985.
12. Storch GA, Myers N: Latex-agglutination test for rubella antibody: Validity of positive results assessed by response to immunization and comparison with other tests. J Infect Dis 149:459–464, 1984.
13. Vaananen P, Haiva VM, Koskela P, Meurman O: Comparison of a simple latex agglutination test with hemolysisin-gel, hemagglutination inhibition, and radioimmunoassay for detection of rubella virus antibodies. J Clin Microbiol 21:793–795, 1985.
14. Cremer NE, Hagens SJ, Cossen C: Comparison of the hemagglutination inhibition test and an indirect fluorescent-antibody test for detection of antibody to rubella virus in human sera. J Clin Microbiol 11:746–747, 1980.
15. Zartarian MV, Friedly G, Peterson EM, de la Maza LM: Detection of rubella antibodies by hemagglutination inhibition, indirect fluorescent-antibody test, and enzyme-linked immunosorbent assay. J Clin Microbiol 14:640–645, 1981.
16. Abbott GG, Safford JW, MacDonald RG, Craine MC, Applegren RR: Development of automated immunoassays for immune status screening and serodiagnosis of rubella virus infection. J Virol Methods 27:227–240, 1990.
17. Hossain A, Ramia S, Bakir TMF: Comparison of hemagglutination test, enzyme-linked immunosorbent assay and indirect immunofluorescence antibody test for determination of rubella immune status. J Trop Med Hyg 91:216–221, 1988.
18. Schaefer LE, Dyke JW, Meglio FD, Murray PR, Crafts W, Niles AC: Evaluation of microparticle enzyme immunoassays for immunoglobulins G and M to rubella virus and *Toxoplasma gondii* on the Abbott IMx automated analyzer. J Clin Microbiol 27:2410–2413, 1989.
19. Skurrie IJ, Head IL, Garland SM: Detection of rubella-specific immunoglobulin G: Comparison of the enzyme-linked immunosorbent assay and an automated microparticle enzyme immunoassay (IMx). J Clin Microbiol 29:1752–1753, 1991.
20. Rubella prevention. MMWR 30:37–47, 1981.
